# Neutrophil-lymphoycyte-ratio, platelet-lymphocyte-ratio and procalcitonin for early assessment of prognosis in patients undergoing VA-ECMO

**DOI:** 10.1038/s41598-021-04519-7

**Published:** 2022-01-11

**Authors:** Sebastian Roth, René M’Pembele, Alexandra Stroda, Catrin Jansen, Giovanna Lurati Buse, Udo Boeken, Payam Akhyari, Artur Lichtenberg, Markus W. Hollmann, Ragnar Huhn, Hug Aubin

**Affiliations:** 1 Department of Anesthesiology, Medical Faculty and University Hospital Duesseldorf, Heinrich-Heine-University Duesseldorf, Moorenstr. 5, 40225 Duesseldorf, Germany; 2 Department of Cardiac Surgery, Medical Faculty and University Hospital Duesseldorf, Heinrich-Heine-University Duesseldorf, Moorenstr. 5, 40225 Duesseldorf, Germany; 3 Department of Anesthesiology, Amsterdam University Medical Center (AUMC), Location AMC, Meibergdreef 9, 1105 AZ Amsterdam, The Netherlands

**Keywords:** Cardiac device therapy, Prognostic markers

## Abstract

The use of veno-arterial extracorporeal membrane oxygenation (VA-ECMO) is increasing, but mortality remains high. Early assessment of prognosis is challenging and valid markers are lacking. This study aimed to investigate Neutrophil–Lymphocyte Ratio (NLR), Platelet-Lymphocyte-Ratio (PLR) and Procalcitonin (PCT) for early assessment of prognosis in patients undergoing VA-ECMO. This retrospective single-center cohort study included 344 consecutive patients ≥ 18 years who underwent VA-ECMO due to cardiogenic shock. Main exposures were NLR, PLR and PCT measured within 24 h after VA-ECMO initiation. The primary endpoint was all-cause in-hospital mortality. In total, 92 patients were included into final analysis (71.7% male, age 57 ± 14 years). In-hospital mortality rate was 48.9%. Receiver operating characteristics (ROC) curve revealed an area under the curve (AUC) of 0.65 [95% confidence interval (CI) 0.53–0.76] for NLR. The AUCs of PLR and PCT were 0.47 [95%CI 0.35–0.59] and 0.54 [95%CI 0.42–0.66], respectively. Binary logistic regression showed an adjusted odds ratio of 3.32 [95%CI 1.13–9.76] for NLR, 1.0 [95%CI 0.998–1.002] for PLR and 1.02 [95%CI 0.99–1.05] for PCT. NLR is independently associated with in-hospital mortality in patients undergoing VA-ECMO. However, discriminative ability is weak. PLR and PCT seem not to be suitable for this purpose.

## Introduction

Veno-arterial extracorporeal membrane oxygenation (VA-ECMO) is supposed to support patients suffering from refractory cardiogenic shock^1–3^. The use of VA-ECMO has increased in recent years^3,4^. However, mortality of these patients is still high with mortality rates of approximately 50%^4,5^. One of the most important issues in terms of treating VA-ECMO patients is to understand which patients really benefit from VA-ECMO. The initiation of VA-ECMO is often done in emergency situations so that treating physicians do not have a lot of time to select suitable patients. Therefore, once initiated, the assessment of prognosis and the decision about therapy futility is even more crucial. Several scores such as the SAVE-score have been suggested in this context^6^, but to date, valid markers are lacking.

Typical complications of patients undergoing VA-ECMO include bleeding, major adverse cardiovascular events (MACE), thromboembolic complications, acute kidney injury or acute liver failure^2,7,8^. Another factor that has been shown to be associated with mortality in patients with VA-ECMO is systemic inflammation^9^. To quantify systemic inflammation, several inflammation markers are available. Calculated from white blood cell count, the Neutrophil–Lymphocyte Ratio (NLR) and the Platelet-Lymphocyte-Ratio (PLR) are two new markers of systemic inflammation which are easily available^10–12^. Previous studies revealed that elevated NLR and PLR are independently associated with all-cause mortality and cardiovascular disease in diverse cohorts^11–13^. In patients with VA-ECMO, data on the prognostic value of NLR and PLR are scarce. Another marker of systemic inflammation is Procalcitonin (PCT) which is regularly used in intensive care medicine, e.g. as a marker of sepsis^14^. However, the value of PCT in terms of early prognosis of VA-ECMO patients is also still underexplored. Against this background, we aimed to investigate NLR, PLR and PCT to predict in-hospital mortality and compared discriminative ability and independent association of these markers.

## Methods

We conducted a retrospective, single-center cohort study in accordance with the guidelines for good clinical practice (GCP) and the declaration of Helsinki. The study was approved by the ethical committee of the Heinrich-Heine-University, Duesseldorf, Germany (reference number 5141R). All included patients are registered in the local VA-ECMO database and gave written informed consent for registration in advance. This manuscript follows the Strengthening the Reporting of Observational Studies in Epidemiology (STROBE) guidelines for retrospective cohort studies.

### Participants

344 patients ≥ 18 years of age treated with VA-ECMO due to refractory cardiogenic shock between 2011 and 2018 at the University hospital Duesseldorf, Germany were included. All VA-ECMO systems were temporary devices. Exclusion criteria were missing values for NLR, PLR or PCT or inconclusive medical records regarding the primary endpoint.

### Main exposures

The main exposures of this study were NLR, PLR and PCT. The rationale to choose NLR and PLR was based on the promising results in other settings^11,12^. PCT was chosen to include one established marker of systemic inflammation as comparison that may also be useful in patients with cardiac disease^15^. All markers were measured within 24 h after VA-ECMO initiation. If multiple values were available, first measurement after initiation was chosen. White blood cell count was measured via automated laboratory devices. NLR was calculated by dividing absolute neutrophils to absolute lymphocytes as calculated from automated white blood cell count. PLR was calculated in the same manner using absolute platelets and absolute lymphocytes. PCT values were extracted as determined by the local laboratory. As white blood cell count is routinely measured only once a week in our center, a relevant number of missing NLR and PLR values could be expected, but was considered acceptable as no selection bias could be identified. We also calculated Sequential Organ Failure Assessment (SOFA) score at the same time point (= within 24 h after VA-ECMO initiation) as additional exposure.

### Outcome assessment and data collection

The primary endpoint of this study was all-cause in-hospital mortality. Data for this endpoint and all further patient characteristics were extracted from patients medical records by trained personnel of the study team.

### Sample size and choice of covariables

As we conducted a retrospective exploratory data analysis, formal sample size calculation was not implemented. However, based on the current literature, we expected an all cause in-hospital mortality for patients treated with VA-ECMO of 50%^4,6^. A sample size of 92 patients meeting the inclusion and exclusion criteria was available. Thus, we expected approximately 45–50 events in total which allowed multivariable adjustment including up to five co-variables^16^. These co-variables were predefined and included age^17^, pre-existing coronary artery disease^18^, duration of VA-ECMO therapy^19^ and continous veno-venous hemodialysis (CVVHD)^20^. The choice of these covariables was driven by the fact that all variables have been shown to be associated with mortality in VA-ECMO patients in the literature as referenced next to each variable. Post-factum, we observed 45 events so that the inclusion of all predefined variables was acceptable.

### Statistical analysis

Complete case analysis was performed. Categorical data are presented as absolute counts (percent). Continuous data are presented as mean ± standard deviation. Discrimination of NLR, PLR and PCT for in-hospital mortality was analyzed by receiver operating characteristics (ROC) curve and the resulting area under the curve (AUC). A cutoff value for NLR was determined by Youden Index. Cutoff-values for PLR and PCT were not calculated as there was no discrimination according to ROC curve. To quantify the independent association of NLR, PLR and PCT with in-hospital mortality, multivariate binary logistic regression with forced entry of the predefined covariables was conducted. For NLR, data-driven cutoff was used. Net reclassification index (NRI) and integrated discrimination index (IDI) were calculated for all three biomarkers. De Long-test was performed to compare AUCs of ROC-curves. A p-level < 0.05 was considered significant. We performed the same statistical procedure with SOFA score (including ROC analysis and multivariate logistic regression) and evaluated the prognostic value of NLR when added to SOFA score by comparing the AUCs of a logistic regression model including SOFA score with and without NLR using De Long test.

### Ethics approval and consent to participate

This retrospective cohort study was approved by the ethical committee of the Heinrich-Heine-University, Duesseldorf, Germany (reference number 5141R).

## Results

**Figure 1:**
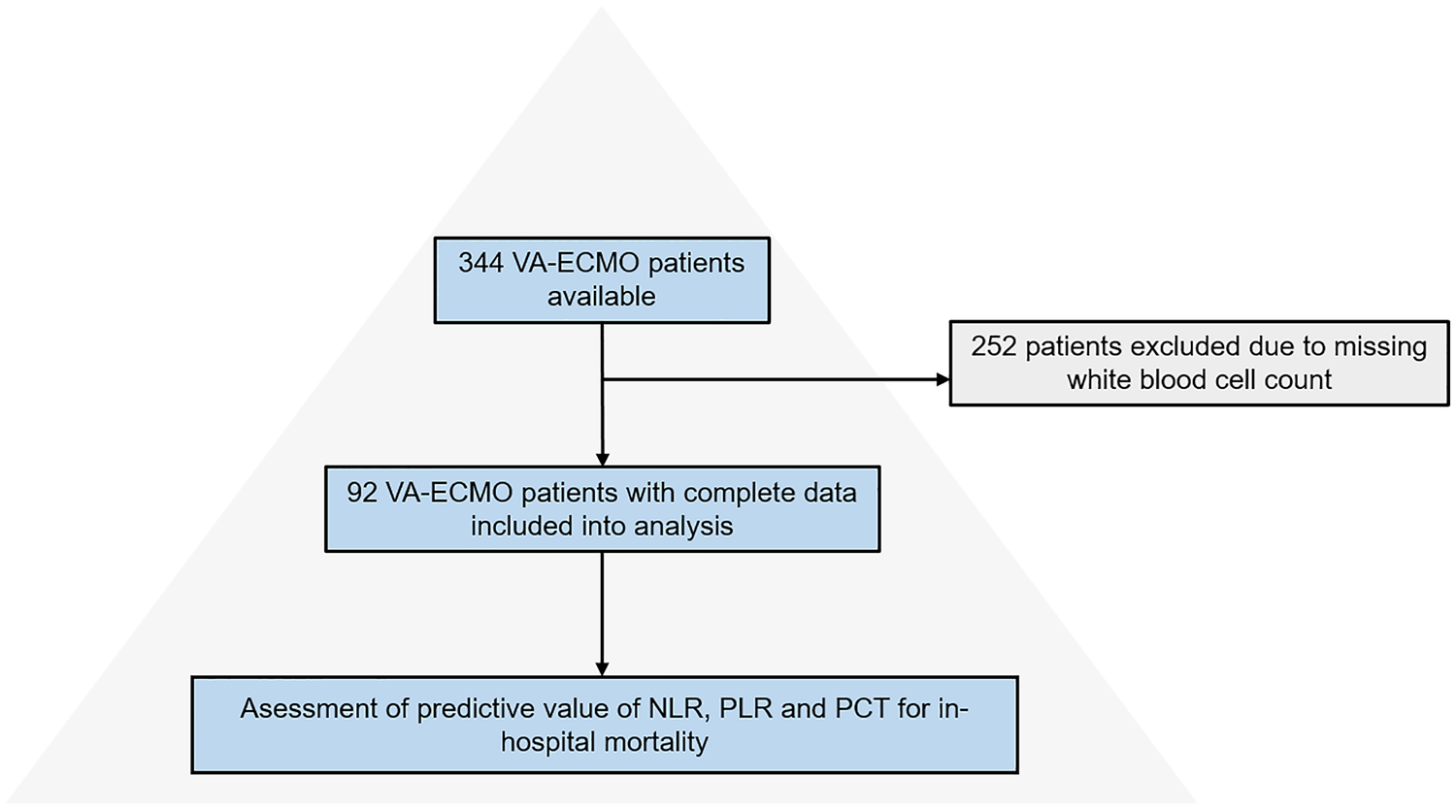
Study flow chart showing selection process of the study cohort. *VA-ECMO* veno-arterial membrane oxygenation, *NLR* neutrophil–lymphocyte-ratio, *PLR* platelet-lymphocyte-ratio, *PCT* procalcitonin.

In total, 344 patients were screened for this study. As white blood cell count was routinely measured only once a week, 252 patients had to be excluded due to missing values. Consequently, 92 patients with complete data remained for statistical analysis (see Fig. 1). 71.7% patients were male and mean age 57 ± 14 years. In-hospital mortality rate was 48.5%. Detailed patient characteristics are presented in Table 1.

**Table 1:** Patient characteristics.

	N (%)	Mean (± SD)
Baseline characteristics	66 (71.7%)	
Male sex no. (%)		
Age (years)		57 ± 14
**Indications for VA-ECMO**		
Post-cardiotomy	36 (39.1%)	
Acute myocardial infarction	21 (22.8%)	
Cardiopulmonary resuscitation	11 (12%)	
Other reasons of cardiogenic shock	24 (26.1%)	
**Comorbidities**		
Coronary artery disease	57 (62%)	
History of myocardial infarction	45 (48.9%)	
Peripheral artery disease	10 (10.9%)	
History of stroke	9 (9.8%)	
Diabetes mellitus	26 (28.3%)	
Arterial hypertension	36 (39.1%)	
**Inflammatory markers and SOFA score**		
C-reactive protein (mg/dl)		7 ± 12.5
Procalcitonin (ng/ml)		10.7 ± 22
Neutrophil–lymphocyte-ratio (× 1000/ul)		12.2 ± 7.7
Platelet-Lymphocyte-Ratio (× 1000/ul)		244 ± 205
SOFA score		11.4 ± 2.4
**Hospital stay**		
Death in hospital	45 (48.9%)	
Duration of VA-ECMO therapy		9 ± 7
Duration of hospital stay		28 ± 31
Thromboembolic complication	31 (33.7%)	
Major bleeding complication	35 (38%)	
AKI requiring CVVHD	53 (57.6%)	
**Coagulation parameters**		
Fibrinogen (mg/dl)		316 ± 139
Quick (%)		47 ± 20
aPTT (sec)		69 ± 46
D-Dimer (mg/l)		15 ± 24
**Other laboratory parameters**		
Bilirubin (mg/dl)		1.7 ± 1.7
Creatinine (mg/dl)		1.9 ± 1.5
High-sensitive troponin T (ng/l)		5527 ± 12,069
Creatinine kinase (U/l)		1531 ± 2481
Creatinine kinase – MB (U/l)		141 ± 152
Lactate dehydrogenase (U/l)		1090 ± 1329

Data are presented as mean ± standard deviation (SD) or as absolute numbers with percentages.

*SOFA* sequential organ failure assessment, *VA-ECMO* veno-arterial extracorporeal membrane oxygenation, *AKI* acute kidyney injury, *CVVHD* continous veno-venous hemodialysis, *aPTT* activated partial thromboplastin time.

### ROC analysis

**Figure 2:**
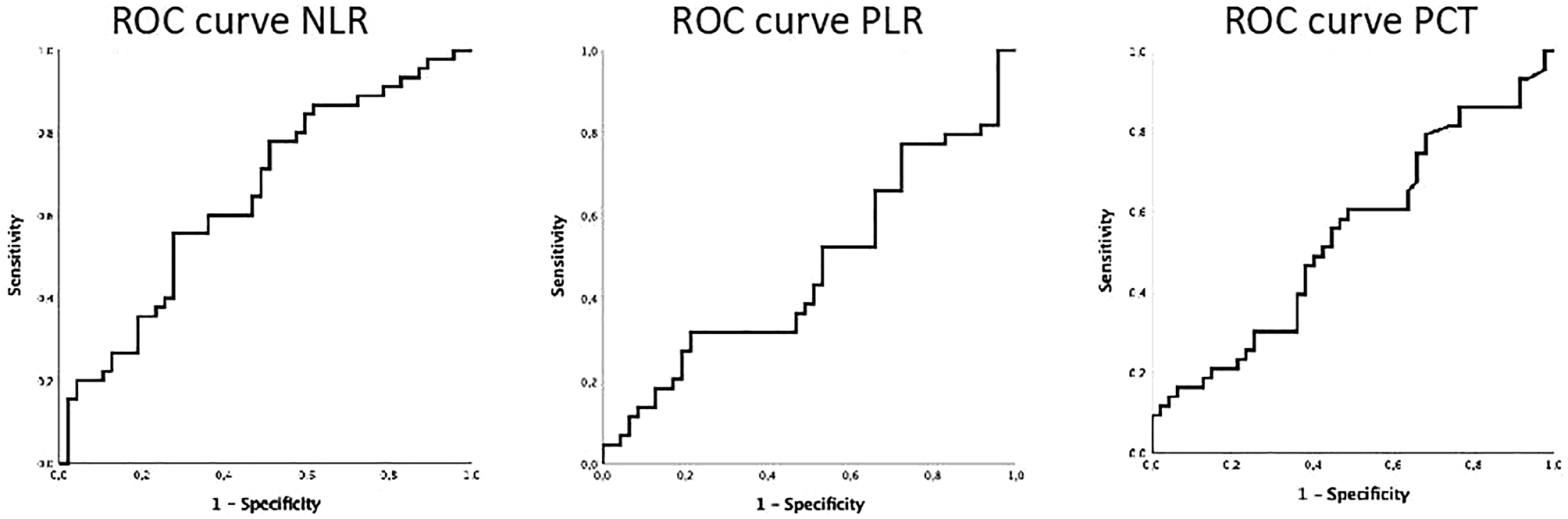
Receiver operating characteristics (ROC) curves showing the discrimination of Neutrophile-Lymphocyte Ratio (NLR), Platelet-Lymphocyte-Ratio (PLR) and Procalcitonin (PCT) for all-cause in-hospital mortality. ROC analysis of NLR revealed an AUC of 0.65 [95% confidence interval (CI) 0.53–0.76; p = 0.015] for NLR. The AUCs of PLR and PCT were 0.47 [95%CI 0.35–0.59; p = 0.645] and 0.54 [95%CI 0.42–0.66; p = 0.521].

ROC analysis of NLR revealed an AUC of 0.65 [95% confidence interval (CI): 0.53–0.76; p = 0.015]. The AUCs of PLR and PCT were 0.47 [95%CI 0.35–0.59; p = 0.645] and 0.54 [95%CI 0.42–0.66; p = 0.521], respectively (see Fig. 2). According to De Long test, AUC of NLR was not significantly higher than AUC of PLR and PCT.

### Youden index

Calculation of Youden index showed a cutoff of 13 for NLR. For PLR and PCT, no cutoff calculation was performed as the discrimination of these values for in-hospital mortality was poor based on ROC analysis.

### Multivariate binary logistic regression

Multivariate binary logistic regression revealed an adjusted odds ratio (OR) of 3.32 [95%CI 1.13–9.76; p = 0.029] for NLR (see Table 2). Adjusted ORs for PLR and PCT were 1.0 [95%CI 0.998–1.002; p = 0.951] and 1.02 [95%CI 0.99–1.05; p = 0.093], respectively (see Tables 3 and 4). Next to NLR, CVVHD was the only covariable that showed an independent association with in-hospital-mortality in all three logistic regression models (p < 0.0001).

**Table 2:** Multivariate binary logistic regression—neutrophil–lymphocyte-ratio.

Variable	Regression coefficient	Odds ratio	95% Confidence interval	p-value
NLR cutoff	1.2	3.32	1.13–9.76	**0.029**
Age	0.01	1.01	0.97–1.05	0.74
Coronary artery disease	0.57	1.78	0.55–5.69	0.334
Days of VA-ECMO therapy	0.04	1.04	0.96–1.12	0.346
CVVHD	2.2	8.99	3.01–26.28	** < 0.0001**

Significant values are in bold.

*NLR* neutrophile lymphocyte ratio, *VA-ECMO* veno-arterial extracorporeal membrane oxygenation, *CVVHD* continous veno-venous hemodialysis.

**Table 3:** Multivariate binary logistic regression—platelet-lymphocyte-ratio.

Variable	Regression coefficient	Odds ratio	95% Confidence interval	p-value
PLR	0.0	1.0	0.998–1.002	0.951
Age	0.02	1.02	0.98–1.06	0.31
Coronary artery disease	0.23	1.26	0.42–3.84	0.681
Days of VA-ECMO therapy	0.04	1.04	0.96–1.12	0.336
CVVHD	2.15	8.59	3.05–24.25	** < 0.0001**

Significant values are in bold.

*PLR* platelet-lymphocyte-ratio, *VA-ECMO* veno-arterial extracorporeal membrane oxygenation, *CVVHD* continous veno-venous hemodialysis.

**Table 4:** Multivariate binary logistic regression—procalcitonin.

Variable	Regression coefficient	Odds ratio	95% Confidence interval	p-value
Procalcitonin	0.02	1.02	0.996–1.053	0.093
Age	0.03	1.03	0.99–1.07	0.188
Coronary artery disease	0.23	1.25	0.4–3.89	0.696
Days of VA-ECMO therapy	0.03	1.03	0.94–1.12	0.542
CVVHD	2.25	9.53	3.22–28.18	** < 0.0001**

Significant values are in bold.

*VA-ECMO* veno-arterial extracorporeal membrane oxygenation, *CVVHD* continous veno-venous hemodialysis.

### Net reclassification index and integrated discrimination index

Overall NRI of NLR was 19.6%. NRI of NLR for events was 6.8% [95%CI 1.4–18.7%] and NRI of NLR for non-events was 12.8% [95%CI 4.8–25.7%]. Overall NRI of PLR was − 7% (NRI events = − 7% [95%CI − 19 to 1.5%]; NRI non-events = 0%) and overall NRI of PCT was 8.8% (NRI events = 2.4% [95%CI 0.1–12.6%]; NRI non-events = 6.4% [95%CI 1.3–17.5%]. IDI of NLR, PLR and PCT was 3.6%, 0% and 1.5%, respectively.

### ROC analysis and multivariate logistic regression for SOFA score

Mean SOFA score measured within 24 h after initiation of VA-ECMO therapy was 11.4 ± 2.4. ROC analysis for SOFA score and in-hospital mortality revealed an AUC of 0.58 [95%CI 0.46–0.70; p = 0.182] (see Supplementary Fig. 1). According to multivariate binary logistic regression, there was no significant association between SOFA score and in-hospital mortality (adjusted OR: 1.01 [95%CI 0.81–1.25; p = 0.967] (see Supplementary Table 1). The AUC of the whole model was 0.8 [95%CI 0.71–0.89; p < 0.0001]. The AUC improved to 0.83 [95%CI 0.74–0.91; p < 0.0001] when NLR was added to this model. This improvement was not significantly different based on De Long test (p = 0.365).

## Discussion

In this study, we found out that NLR is independently associated with in-hospital mortality in patients undergoing VA-ECMO. In addition, we could show that there was no association for PLR and PCT. The discriminative ability of NLR, PLR and PCT was weak.

According to the current literature, systemic inflammation plays a key role in the complex pathophysiology of critical illness and there is good evidence that systemic inflammation is associated with worse outcome in patients undergoing VA-ECMO^9^. In the following, we will discuss existing knowledge regarding the predictive value of NLR, PLR and PCT in VA-ECMO patients. In addition, we will state what our results do add to the literature.

To date – to our best knowledge—there is only one study by Yost and colleagues that investigated the predictive value of NLR in adult patients supported by VA-ECMO^21^. This study included 107 retrospective patients who underwent VA-ECMO implantation for cardiogenic shock and reported that NLR is suitable as a prognostic marker for survival in this cohort. Unfortunately, the authors only performed a comparison of mean NLR values and neither ROC analysis nor multivariate analysis was done. The results of our study add to these limited data by showing that after multivariable adjustment, NLR is still associated with an increased in-hospital mortality (OR = 3.32 [95%CI 1.13–9.76; p = 0.029]). In addition, we defined the first potential cutoff value for NLR in VA-ECMO patients which was 13 according to Youden Index. This cutoff is higher than the existings cutoffs of NLR in the noncardiac surgery setting (e.g. NLR cutoff = 4)^11^, but with regard to the extent of systemic inflammation that is caused by cardiogenic shock and VA-ECMO itself, higher cutoff seems plausible in this setting. Furthermore, we could show that the AUC of a logistic regression model including SOFA score (and our 4 predefined variables) could be improved when NLR was added to this model. Although this improvement was not significantly different, our findings clarify that NLR should be considered as early prognostic marker.

While literature on NLR and VA-ECMO is scarce, there are some studies that analyzed the relevance and prognosis of other leukocyte profiles. For example, Siegel et al. prospectively investigated 22 VA-ECMO patients and 15 control subjects in terms of monocyte dysfunction and found out that this pathology could be suitable to predict mortality in patients undergoing VA-ECMO^22^.

Referring to PLR, this is the first study that investigated this marker in adult VA-ECMO patients. There is only one study by Arslanoglu and colleagues that assessed the prognostic value of PLR in 67 pediatric cardiac surgery patients with VA-ECMO^23^. This study found out that there was no significant relationship between PLR and various postoperative blood parameters and blood gas values. Our results add to these data and show that PLR seems not to be suitable to predict in-hospital mortality as discrimination in ROC analysis was poor. As the sample size in our study was rather small, these results are not sufficient to draw final conclusions. However, based on our data we can say that NLR seems to be superior to PLR in this context so that physicians might rather have a look on NLR than PLR if both values are available.

In terms of PCT, several studies are available that investigated the predictive value of this marker in cardiac surgery and most of these studies came to the conclusion that PCT is a valuable prognostic marker in this setting^24^. In patients undergoing VA-ECMO, literature is very limited. Do Wan Kim and co-authors found out in 38 adult cardiogenic shock patients that a PCT level > 10 ng/mL during VA-ECMO treatment was significantly associated with increased in-hospital mortality (p < 0.01)^25^. Although multivariate analysis was performed, the authors could only adjust for age and nosocomial infections. Most other studies concentrated on the association between PCT and infections, but did not assess the predictive value in terms of mortality. Our study therefore adds new knowledge by showing that the discrimination of PCT for in-hospital-mortality in VA-ECMO patients seems to be poor.

This study has several limitations. The main limitation is the huge amount of missing data. As explained above, this is mainly due to the fact that white blood cell count is routinely measured only once a week in our center. This measurement is mostly performed on a specific week day and does not depend on patient- or procedure-related factors so that we concluded that no or only low selection bias should be present. Another limitation is the retrospective design. However, mortality rate in this study corresponds to the current literature so that we assume that our study cohort can be regarded as representative. Finally, this study did only analyze three inflammatory markers, although other markers (e.g. Interleukin-6) are available that might also be investigated in this context.

## Conclusions

In conclusion, this study showed that NLR is independently associated with in-hospital mortality in patients with VA-ECMO. However, the discriminative ability of NLR is weak and the sample size of our cohort was small so that further studies with a prospective design and larger cohorts are needed to reevaluate our findings. According to our limited data, PLR and PCT seem not to be suitable for assessment of prognosis in patients with VA-ECMO.

## Supplementary Information

Supplementary Information.

## Data Availability

The datasets generated during and/or analysed during the current study are available from the first author on reasonable request.
