# Supplementary material for: Neutrophil-lymphoycyte-ratio, platelet-lymphocyte-ratio and procalcitonin for early assessment of prognosis in patients undergoing VA-ECMO
Source: Sci Rep. 2022 Jan 11;12:542. doi: 10.1038/s41598-021-04519-7 (PMC8752603; doi:10.1038/s41598-021-04519-7)
Supplement: Supplementary file 1 — Supplementary Information. [file 41598_2021_4519_MOESM1_ESM.docx]

**Supplementary material:**


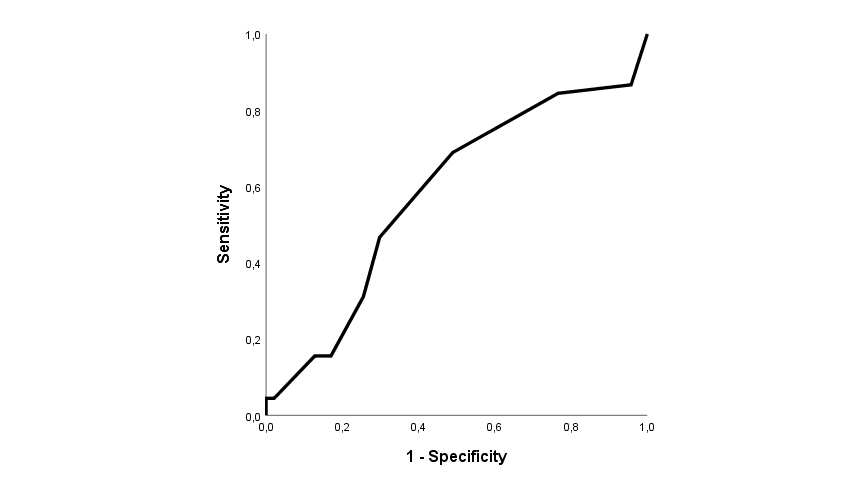


**Supplementary Figure 1**: ROC analysis for SOFA score and in-hospital mortality revealed an AUC of 0.58 [95%CI: 0.46-0.70; p=0.182].

**Supplementary Table 1:** Multivariate binary logistic regression including SOFA score

| **Variable** | **Regression coefficient** | **Odds ratio** | **95% Confidence interval** |  | **p-value** |
| --- | --- | --- | --- | --- | --- |
| SOFA score | 0.01 | 1.01 | 0.81-1.25 |  | 0.967 |
| Age | 0.20 | 1.02 | 0.98-1.06 |  | 0.315 |
| Coronary artery disease | 0.27 | 1.31 | 0.42-4.04 |  | 0.643 |
| Days of VA-ECMO therapy | 0.04 | 1.04 | 0.96-1.12 |  | 0.336 |
| CVVHD | 2.17 | 8.78 | 3.05-25.29 |  | **<0.0001** |

SOFA = Sequential Organ Failure Assessment; VA-ECMO = Veno-Arterial Extracorporeal Membrane Oxygenation; CVVHD = Continous Veno-Venous Hemodialysis
